# Correction: Castration delays epigenetic aging and feminizes DNA methylation at androgen-regulated loci

**DOI:** 10.7554/eLife.92968

**Published:** 2023-10-13

**Authors:** Victoria J Sugrue, Joseph Alan Zoller, Pritika Narayan, Ake T Lu, Oscar J Ortega-Recalde, Matthew J Grant, C Simon Bawden, Skye R Rudiger, Amin Haghani, Donna M Bond, Reuben R Hore, Michael Garratt, Karen E Sears, Nan Wang, Xiangdong William Yang, Russell G Snell, Timothy A Hore, Steve Horvath

**Keywords:** Human, Mouse, Other

Sugrue VJ, Zoller JA, Narayan P, Lu AT, Ortega-Recalde OJ, Grant MJ, Bawden CS, Rudiger SR, Haghani A, Bond DM, Hore RR, Garratt M, Sears KE, Wang N, Yang XW, Snell RG, Hore TA, Horvath S. 2021. Castration delays epigenetic aging and feminizes DNA methylation at androgen-regulated loci. *eLife* **10**:e64932. doi: 10.7554/eLife.64932.

Published 6 July 2021

We were notified via PubPeer of an error in Figure 5. In panel C, the ChIP-seq data for AR Prostate Epithelium was duplicated resulting in identical tracks presented for AR Prostate Epithelium and AR Breast. Upon examining other figures for similar issues, we found duplication in Figure 5 Supplement 1, panel B, where the AR Prostate Epithelium track was again shown in place of FOXP1 Prostate Epithelium.

We predict that these duplications occurred while selecting tracks from the Cistrome database to display on the UCSC genome browse, which retains tracks from previous searches in the viewer regardless of repetition. The inclusion of identical tracks in these figures was an unintentional oversight.

We have since retrieved the correct AR Breast and FOXP1 Prostate Epithelium data and replaced the duplicated tracks.

The corrected Figure 5 is shown here:

**Figure 1:**
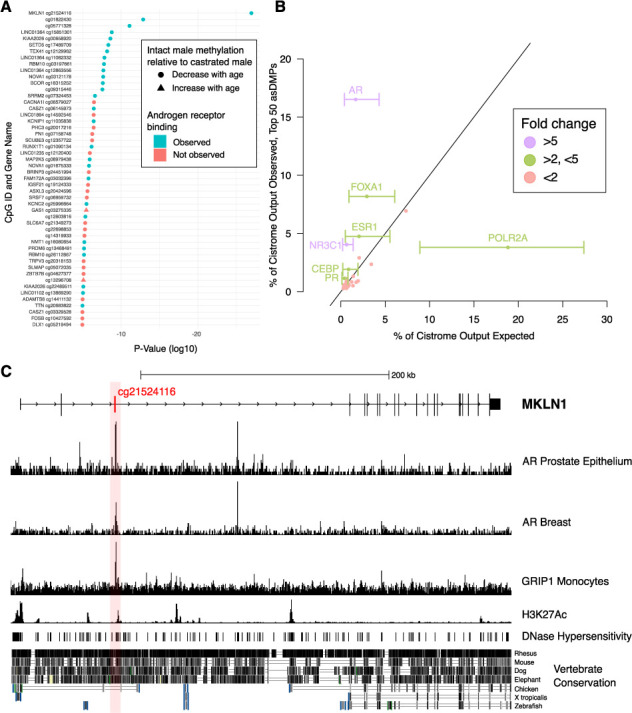


The originally published Figure 5 is shown for reference:

**Figure 2:**
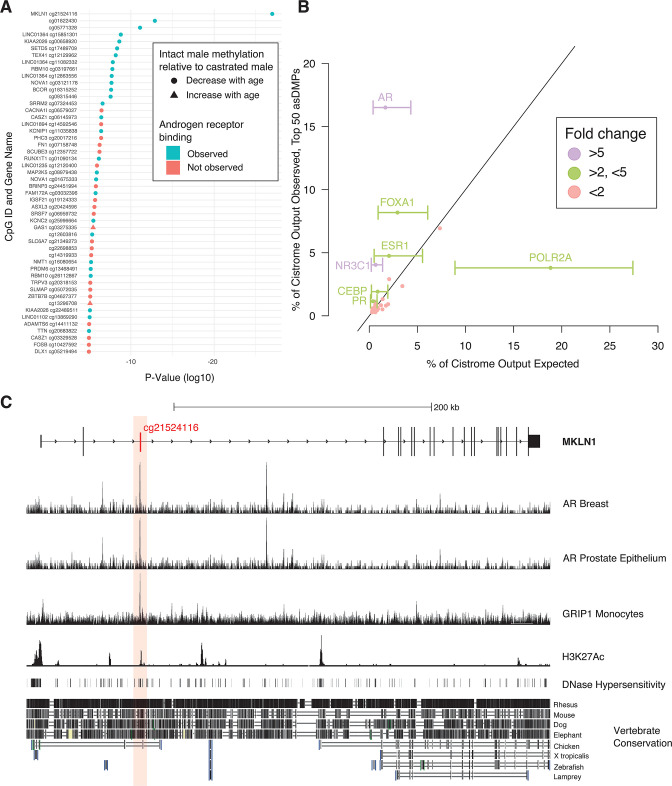


The corrected Figure 5 Supplement 1 is shown here:

**Figure 3:**
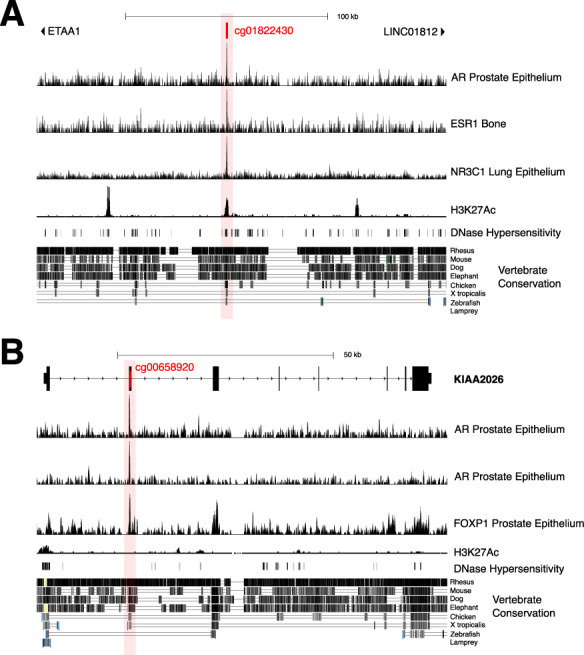


The originally published Figure 5 Supplement 1 is shown for reference:

**Figure 4:**
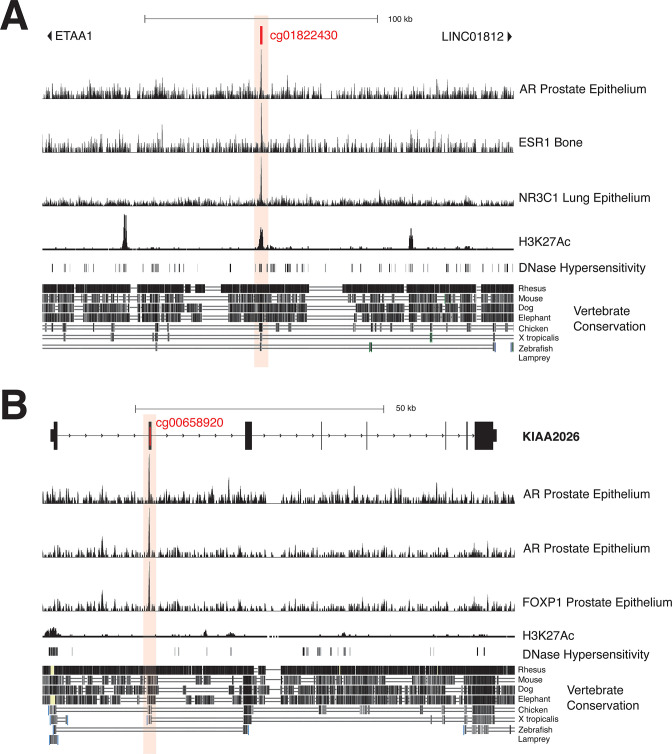


We thank the users of PubPeer for alerting us of this issue. The article has been corrected accordingly.

